# Integrating Advanced Metabolomics and Machine Learning for Anti-Doping in Human Athletes

**DOI:** 10.3390/metabo15110696

**Published:** 2025-10-27

**Authors:** Mohannad N. AbuHaweeleh, Ahmad Hamdan, Jawaher Al-Essa, Shaikha Aljaal, Nasser Al Saad, Costas Georgakopoulos, Francesco Botre, Mohamed A. Elrayess

**Affiliations:** 1 College of Medicine, QU Health, Qatar University, Doha P.O. Box 2713, Qatar; ma1908120@qu.edu.qa (M.N.A.);; 2 Qatar Antidoping Agency, Doha P.O. Box 120, Qatar; jawaher@qad.qa (J.A.-E.); shaikha@qad.qa (S.A.); nasser@qad.qa (N.A.S.); 3 Saudi Anti-Doping Laboratory, Department of Pathology and Laboratory Medicine (MC-1122), Ministry of National Guard Health Affairs, King Abdulaziz Medical City, Building 6419 Ar Rimaya, Unit 1, P.O. Box 22490, Riyadh 14611-2860, Saudi Arabia; costas@mngha.med.sa; 4 Department of Chemistry ‘Ugo Schiff’, University of Florence, 50139 Florence, Italy; francesco.botre@unifi.it; 5 Biomedical Research Center, QU Health, Qatar University, Doha P.O. Box 2713, Qatar

**Keywords:** anti-doping, metabolomics, machine learning, artificial intelligence, doping detection, athlete biological passport, biomarkers, predictive modeling, data analytics, targeted metabolomics, untargeted metabolomics, mass spectrometry, UHPLC, NMR spectroscopy, sports integrity

## Abstract

The ongoing challenge of doping in sports has triggered the adoption of advanced scientific strategies for the detection and prevention of doping abuse. This review examines the potential of integrating metabolomics aided by artificial intelligence (AI) and machine learning (ML) for profiling small-molecule metabolites across biological systems to advance anti-doping efforts. While traditional targeted detection methods serve a primarily forensic role—providing legally defensible evidence by directly identifying prohibited substances—metabolomics offers complementary insights by revealing both exogenous compounds and endogenous physiological alterations that may persist beyond direct drug detection windows, rather than serving as an alternative to routine forensic testing. High-throughput platforms such as UHPLC-HRMS and NMR, coupled with targeted and untargeted metabolomic workflows, can provide comprehensive datasets that help discriminate between doped and clean athlete profiles. However, the complexity and dimensionality of these datasets necessitate sophisticated computational tools. ML algorithms, including supervised models like XGBoost and multi-layer perceptrons, and unsupervised methods such as clustering and dimensionality reduction, enable robust pattern recognition, classification, and anomaly detection. These approaches enhance both the sensitivity and specificity of diagnostic screening and optimize resource allocation. Case studies illustrate the value of integrating metabolomics and ML—for example, detecting recombinant human erythropoietin (r-HuEPO) use via indirect blood markers and uncovering testosterone and corticosteroid abuse with extended detection windows. Future progress will rely on interdisciplinary collaboration, open-access data infrastructure, and continuous methodological innovation to fully realize the complementary role of these technologies in supporting fair play and athlete well-being.

## 1. Introduction

The ongoing issue of doping in sports undermines the integrity of athletic competition and challenges the efficacy of existing regulatory frameworks, particularly given the continual emergence of novel prohibited substances and sophisticated doping methods that complicate detection efforts [1]. Anti-doping efforts in sports were catalyzed by critical incidents, originating back to the 1960 Olympic Games due to the death of a Danish cyclist, highlighting the urgent need for a structured and proactive framework to deter the use of prohibited substances and safeguard athlete health [2]. Despite the establishment of dedicated anti-doping bodies, such as the World Anti-Doping Agency (WADA), National Anti-Doping Organizations (NADOs), Major Event Organizations, National Olympic Committees (NOCs), and International Federations, and sustained global efforts to promote clean sport, athletes and their support teams continue to adopt increasingly sophisticated strategies to evade detection. The persistent prevalence of doping has been partly attributed to challenges in policy implementation and variability in compliance among WADA signatories, undermining the overall effectiveness of anti-doping initiatives [3]. While there is limited data on the prevalence of doping among athletes, a recent study found that the estimated prevalence of doping among elite athletes in the United States ranged between 6.5% and 9.2% [4].

Recognizing these persistent challenges, the World Anti-Doping Code was developed to harmonize anti-doping policies and regulations across sporting authorities worldwide. First introduced by WADA in 2003 and most recently updated in 2021, the Code, together with eight supporting International Standards covering areas such as prohibited substances, athlete testing, results management, and privacy, serves as the backbone of global doping control. It aims to promote consistency, enhance policy implementation, and strengthen compliance among stakeholders, thereby supporting the broader goal of maintaining integrity and fairness in sport.

However, the effectiveness of even the most robust regulatory frameworks ultimately depends on the tools available for enforcement. Traditional detection techniques, such as gas chromatography–mass spectrometry (GC-MS) and liquid chromatography–mass spectrometry (LC-MS), have been instrumental in clinical toxicology [5] and remain foundational for the identification of doping substances [6], though they are often labor-intensive and susceptible to some errors [1]. Additionally, these traditional approaches are also heavily dependent on prior knowledge of drug structures and known metabolic byproducts, limiting their efficacy in scenarios involving endogenous substances or newly synthesized performance-enhancing agents.

In response to these challenges, metabolomics has emerged as a powerful tool in the anti-doping field [6]. Metabolomics, a comprehensive approach that maps the complete set of small molecules in a biological system, offers considerable potential to improve doping detection in sports [7]. Early studies have shown promise in uncovering novel metabolic signatures indicative of prohibited substance use. However, unlike established analytical methods—which can detect synthetic molecules at extremely low concentrations, even after microdosing—the capability of metabolomics to identify such subtle exposures remains uncertain. Moreover, widespread implementation in standard anti-doping protocols faces additional challenges, including significant variation between individuals, issues with sample handling and stability, and gaps in analytical coverage [7]. Despite these limitations, metabolomics could provide valuable biomarkers for both direct and indirect detection of doping, particularly when integrated with longitudinal, personalized monitoring frameworks such as the athlete biological passport (ABP) [7].

Metabolomics, through the comprehensive profiling of small molecules within biological systems [8], provides a holistic and systems-level perspective on biochemical changes associated with doping practices. Unlike conventional methods that primarily target specific exogenous compounds, metabolomic approaches can detect subtle alterations in endogenous metabolic pathways [8], offering indirect but highly informative evidence of physiological disruption indicative of performance-enhancing substance use. This capability is particularly useful in cases where the drug itself may no longer be present but has left a lingering metabolic footprint. High-throughput techniques such as ultra-high-performance liquid chromatography coupled with high-resolution mass spectrometry (UHPLC-HRMS) and nuclear magnetic resonance (NMR) spectroscopy facilitate the comprehensive analysis of thousands of metabolites in a single sample [9], opening up the door to more nuanced and inclusive anti-doping strategies.

However, the complexity and volume of data generated by metabolomic analyses necessitate advanced computational tools for meaningful interpretation. This is where artificial intelligence (AI), and more specifically machine learning (ML), offers a transformative solution [1]. The application and integration of AI algorithms offer substantial potential to improve the efficiency of anti-doping measures and promote fairness in athletic competitions [10]. While AI can be applied to metabolomic datasets, it is equally valuable for other anti-doping tools such as the ABP [10], a personalized digital record that tracks biological markers in an athlete over time to identify potential doping [11]. Rather than relying solely on detecting banned substances, it monitors changes in specific biomarkers—such as hormone levels—to spot unusual variations that may indicate doping. By focusing on long-term patterns and individual baselines, the ABP enhances the accuracy and effectiveness of anti-doping strategies [11].

ML excels at uncovering complex, nonlinear patterns in high-dimensional datasets—precisely the type of data produced by metabolomics. The use of ML for data analysis, pattern recognition, and model development is increasingly widespread across various disciplines [12]. ML techniques are applied to classify, regress, or cluster complex metabolomic datasets [12]. A typical metabolomic workflow incorporates fundamental ML methods such as support vector machines (SVMs), decision trees, random forests (RFs), neural networks (NNs), and deep learning (DL) to effectively analyze and interpret metabolomic data [12]. Continued advancements in instrumental analytical techniques and ML methodologies are facilitating the development of rapid, scalable, and highly efficient doping detection approaches, thereby strengthening efforts to uphold fairness and integrity within competitive sports [1]. Algorithms such as SVMs, RFs, principal component analysis (PCA), and deep NN can be employed to identify metabolomic signatures that differentiate doped from clean samples. For instance, supervised learning algorithms can be trained using metabolomic profiles from athletes with confirmed erythropoietin (EPO) use, enabling the identification of characteristic metabolic signatures associated with doping. Furthermore, the integration of ML with untargeted metabolomics enables the prediction and identification of previously unknown metabolites, facilitating the discovery of durable biomarkers for doping detection [1].

Beyond classification, unsupervised learning techniques offer additional advantages. Clustering and anomaly detection algorithms can help identify athletes whose metabolic profiles deviate significantly from their historical baselines [13]. This approach could form the basis for individualized monitoring protocols, wherein each athlete’s unique metabolomic fingerprint is tracked over time to detect abnormal shifts. Such a system would mirror and enhance the ABP [14] by adding a layer of metabolic data to hematological and steroidal markers, further increasing its robustness. Moreover, ML can assist in biomarker discovery by pinpointing key features within complex datasets that are most predictive of doping activity, potentially leading to the development of new diagnostic tests and monitoring tools [1,15]. The integration of metabolomics and ML marks a transformative shift in anti-doping science, necessitating interdisciplinary collaboration among analytical chemists, data scientists, sport medicine clinicians, and regulatory authorities, to ensure data accuracy, algorithm reliability, clinical relevance, and ethical compliance. Standardized protocols and the development of large, diverse, and accessible metabolomic datasets will be critical to creating robust, generalizable machine learning models for effective doping detection.

The fight against doping in sports demands a bold reimagining of traditional strategies. The integration of AI and ML into doping detection in sports offers significant advancements over traditional methods, primarily through enhanced accuracy, specificity, and rapid detection capabilities [16]. By harnessing the power of untargeted metabolite profiling and AI-driven data interpretation, anti-doping efforts can move from reactive detection toward proactive and individualized surveillance. While challenges remain, the synergy between these disciplines holds the potential to safeguard the integrity of competitive sports and protect the health and fairness of athletes worldwide [16]. This review explores how metabolomics and ML intersect in anti-doping science.

## 2. Metabolomics in Anti-Doping

While metabolomics is central to this section, it is important to situate it within the current ABP framework. Today’s ABP is built on the hematological and steroidal modules (with an additional endocrine module now operational), which are based on targeted, predefined biomarker panels rather than untargeted metabolomics [17,18,19]. The steroidal module monitors urinary endogenous anabolic androgenic steroid (EAAS) markers and ratios (e.g., Testosterone/Estrogen and related metabolites) as specified in WADA’s EAAS Technical Document [20], while the hematological module tracks blood variables indicative of blood manipulation [19]. These approaches are not metabolomic in design. In contrast, the endocrine module longitudinally monitors growth-hormone-sensitive markers (IGF-1 and P-III-NP) using the GH-2000 score; although still targeted, this multi-analyte endocrine profiling is conceptually closer to metabolomics and has entered routine ABP operations [21,22]. Accordingly, metabolomics in anti-doping should be explicitly framed as largely investigational/adjunct at present rather than a component of the current ABP (except for the endocrine module’s targeted endocrine biomarkers) [22]. This distinction clarifies where metabolomics stands today versus its future potential to complement or inform ABP decision-making as validation and standardization studies are developed [6].

### 2.1. Workflow and Analytical Platforms

#### 2.1.1. Workflow and Selection of Metabolites

Metabolomics workflows in anti-doping include sample collection, preparation, analytical measurement, data analysis, and interpretation [23]. Using various advanced analytical platforms, metabolomics offers comprehensive insights into alterations that indicate doping practices [6]. An integral part of this approach is the combining of targeted and untargeted metabolomics, each with distinct methodologies and advantages, followed by biomarker validation [6,24].

Targeted metabolomics involves quantitative analysis focused on a predefined set of known metabolites, providing high sensitivity and specificity [25]. Due to its highly focused approach, targeted metabolomics allows for precise quantification of metabolites even at very low concentrations, making it particularly effective for detecting subtle biochemical changes associated with doping practices [26,27]. In contrast, untargeted metabolomics involves a qualitative, exploratory analysis aimed at identifying as many metabolites as possible without prior knowledge of their chemical identities [28]. An untargeted approach is particularly beneficial for discovering novel biomarkers that signal illicit substance use, unknown doping agents, or complex metabolic signatures resulting from doping methods such as blood manipulation or hormonal treatments [7]. Untargeted methods offer broad metabolic coverage and increased discovery potential, but they are less accurate compared to the targeted counterpart [29]. Therefore, combining targeted and untargeted metabolomics ensures both precise quantification and extensive metabolic coverage for a specific doping agent such as testosterone [29,30,31]. The SQUAD (Simultaneous Quantitation and Discovery) method integrates targeted and untargeted workflows in a single experiment, allowing for accurate quantification of targeted metabolites and global metabolic profiling [32]. This combined approach addresses limitations of individual methods, providing a more balanced and comprehensive metabolomic analysis. Both targeted and untargeted methods find applications in various fields, including food science, disease research, drug development and detection of doping practices [33].

Following identification of metabolites of interest, biomarker validation is the following step, where candidate biomarkers are examined to determine if they are specific, sensitive, and their utility is reproducible across different populations [34]. Validation employes a pure statistical approach including variable selection methods like PCA, classification models such as partial least squares (OPLS) and logistic regression [35] and receiver operating characteristic (ROC) curves, to evaluate biomarker predictive performance metrics such as specificity and sensitivity [36]. Additionally, examination of metabolites against external validation cohorts and longitudinal sampling is needed to confirm stability and reproducibility over time and across different populations [37]. Proper validation ensures that identified metabolites reliably indicate doping practices rather than physiological variations or other confounding factors [38].

#### 2.1.2. Analytical Platforms and Statistical Analyses Used in Metabolomics

The main analytical platforms used in metabolomics are MS, NMR spectroscopy and LC-MS/MS [39]. LC-MS/MS is the most frequently utilized platform and more convenient to be used for doping agents screening due to its sensitivity, precision, and reproducibility, making it ideal for quantifying low-abundance metabolites [26,40]. Furthermore, LC-MS/MS can discriminate structurally similar metabolites, which adds more specificity and therefore avoiding false-positive results [26].

Gas chromatography–tandem mass spectrometry (GC-MS/MS) remains a mainstay in doping control, particularly for volatile or derivatized steroids and stimulants; routine workflows typically involve enzymatic hydrolysis, liquid–liquid extraction, and trimethylsilylation prior to GC-MS/MS acquisition, enabling high selectivity and low minimal required performance limits (MRPL)-aligned detection limits for numerous Prohibited List analytes [26,41,42]. Gas chromatography–combustion–isotope ratio mass spectrometry (GC-C-IRMS) provides confirmatory evidence for the misuse of pseudoendogenous anabolic steroids (e.g., testosterone) by comparing the carbon isotope ratios (δ^13^C) of target compounds to endogenous reference compounds, and its use is mandated by WADA technical documents when steroidal profiling indicates suspicion [43,44]. Collectively, GC-MS/MS offers broad targeted screening capacity with mature identification criteria, while GC-C-IRMS supplies orthogonal, legally robust confirmation of synthetic origin—together forming a complementary pipeline alongside LC-MS (/MS) and ABP strategies [45,46]. For (semi-)untargeted metabolomics, high-resolution mass spectrometry (e.g., Orbitrap, TOF/QTOF, FT-ICR) is routinely coupled to both LC and GC to enable accurate-mass, MS/MS-rich feature detection and identification, whereas low-resolution triple-quadrupole MS (QQQ) is predominantly used for targeted MRM quantification [45,46,47].

Furthermore, NMR spectroscopy provides complementary advantages, being quantitative, non-destructive, and highly reproducible. It has high capabilities in structural determination and detecting broad metabolic shifts that may indicate doping [48]. However, the lower sensitivity of NMR compared to mass spectrometry platforms limits its utility in identifying metabolites present in minimal amounts [49]. In context of anti-doping, NMR is not used in routine laboratory analyses; instead, its primary application lies in research settings, particularly for elucidating the structures of newly synthesized substances or conducting exploratory metabolic investigations when ample sample material is available.

High-resolution mass spectrometry techniques, such as Orbitrap, Fourier Transform Ion Cyclotron Resonance (FT-ICR/MS), and Quadrupole Time-of-Flight (QTOF-MS), provide accuracy and resolution needed for precise metabolite identification and characterization in anti-doping analyses [50,51]. These platforms are highly suitable for untargeted screening aimed at discovering novel doping biomarkers or unknown metabolites present at very low concentrations [7,51]. Their high resolving power and sensitivity enable detailed metabolic profiling, crucial for differentiating between legitimate physiological variations and doping-induced metabolic alterations, thus significantly improving anti-doping testing effectiveness and accuracy [52,53]. Table 1 compares key metabolomic analytical platforms, emphasizing their suitability for anti-doping applications, advantages in data generation, and inherent challenges.

In addition to analytical performance criteria, WADA technical documents mandate specific statistical approaches to ensure robustness and legal defensibility of results. These include the application of predefined thresholds (e.g., the urinary T/E ratio trigger value of 4:1) and adaptive Bayesian models within the Athlete Biological Passport (ABP) framework [17,54]. Statistical tools such as ROC curves are commonly used to evaluate biomarker discriminative performance [55], while external validation across independent laboratories and cohorts is required to demonstrate robustness and reproducibility [56]. Furthermore, correction for multiple testing, for example through false discovery rate (FDR) adjustment, is essential in metabolomics and consistent with WADA’s requirement to minimize false positives in anti-doping adjudication [57]. In metabolomics workflows, similar principles are applied through data normalization to control batch effects, multivariate statistical methods such as PCA and OPLS for variable selection and dimensionality reduction, and longitudinal modeling approaches that mirror the ABP’s focus on intra-individual variability [20]. While these methods support biomarker discovery and monitoring, metabolomics often operates in a high-dimensional feature space, which can diverge from WADA’s strict requirements for predefined markers, validated thresholds, and regulatory interpretability. Therefore, successful translation of metabolomics into anti-doping depends on balancing exploratory discovery with the statistical rigor and confirmatory frameworks mandated by WADA.

### 2.2. Case Studies

To illustrate the practical value of metabolomics in anti-doping efforts, we highlight several recent studies that demonstrate advanced analytical techniques and their application in detecting substance abuse in athletes.

#### 2.2.1. Salbutamol/Budesonide Abuse Detection

These case studies explore how metabolomics has been applied to detect the misuse of β2 agonists and corticosteroids, particularly salbutamol and budesonide, which are commonly prescribed for respiratory conditions but may be abused for performance enhancement.

Salbutamol (a β2 receptor agonist) and budesonide (a glucocorticoid drug) are both used in the management of various respiratory diseases, such as asthma and chronic obstructive pulmonary disease (COPD) [58]. Their utility lies in their pharmacologic effects, bronchodilation in the case of salbutamol, and anti-inflammatory action in the case of budesonide, which also enhances the airway response to β-adrenergic agonists [59]. These effects are considered desirable in endurance-based sports such as cycling, swimming, and long-distance running. Consequently, there have been reports of salbutamol and budesonide abuse by athletes, necessitating thorough anti-doping screening [60,61]. Detection of use of such compounds was recorded in horse-racing and recently in human athletes [62]. Examples are detection of beta-2 agonists like formoterol and salbutamol, and corticosteroids like budesonide, in biological samples such as urine and serum, and in hair for therapeutic drug monitoring [63,64,65,66]. These methods are often aimed at applications in doping control and clinical management of respiratory diseases.

A targeted drug-metabolism/bioanalytical study by Harps et al. focused on the successful quantification of formoterol, salbutamol, and its metabolite salbutamol-4′-O-sulfate in human urine and serum using UHPLC-MS/MS [64]. Through a randomized-blinded design, different dosages of salbutamol were administered and then samples were collected at the following timepoints: after exercise, 3 h and 24 h after administration. In urine samples, salbutamol reached peak concentrations of 948.5 ng/mL, along with 2738.5 ng/mL of its major metabolite, salbutamol-4′-O-sulfate, following de-glucuronidation. Notable sex-specific differences were observed in both serum levels and urinary excretion. Consistent with its scope, this study demonstrates the utility of targeted UHPLC-MS/MS for quantitative anti-doping bioanalysis rather than metabolomics. In line with the definition used in this review, a metabolomics study would additionally quantify endogenous metabolite perturbations associated with the intervention (e.g., pathway-level changes), which can be performed in parallel with targeted drug/metabolite assays.

Another study performed by Kiss et al. aimed to detect differences in metabolite levels between doped athletes, clean athletes, and non-athletes [65]. This study applied a non-targeted metabolomics approach using two high-resolution mass spectrometry platforms (FT-ICR/MS and UHPLC-QTOF/MS) to analyze urine samples from clean athletes, volunteers, and athletes who tested positive for salbutamol or budesonide. A total of 46 urine samples were analyzed using electrospray ionization in both positive and negative modes, followed by multivariate statistical techniques including PCA and OPLS for group discrimination. The resulting discriminant features were annotated with Mass Spectrometry Translator into Reactions (MassTRIX), a bioinformatics tool designed to analyze and interpret MS-based metabolomics data to map affected metabolic pathways. Salbutamol-treated athletes showed clear metabolic alterations, with salbutamol detected as a strong discriminant signal and associated with changes in amino acid and pentose metabolism. Budesonide also affected particularly steroid hormone biosynthesis, though the statistical model for budesonide was weaker, likely due to smaller sample size or milder metabolic effects. The SUS-plot comparison revealed that while both drugs influenced ABC transporters, salbutamol uniquely impacted unsaturated fatty acid biosynthesis, and budesonide specifically altered arachidonic acid and vitamin B6 pathways. These findings provide a comprehensive view of drug-induced metabolic changes useful for doping control.

A targeted drug-metabolism/bioanalytical study by Deventer and colleagues developed and validated an LC–MS method to quantify budesonide and its major metabolite, 16α-hydroxy-prednisolone, in urine after inhalation [63]. Urine samples from five healthy male volunteers were collected at multiple timepoints (0–48 h) post-inhalation and processed via alkaline liquid–liquid extraction, followed by LC–MS analysis in positive electrospray mode. Calibration, recovery, precision, and specificity were assessed, with method sensitivity reaching 0.5 ng/mL for budesonide and 5 ng/mL for its metabolite. Budesonide itself was undetectable in all urine samples post-inhalation, consistent with its rapid metabolism. In contrast, 16α-hydroxy-prednisolone was measurable in four of five participants, with peak concentrations occurring 1–3 h after administration and remaining detectable for up to 12 h. Cumulative urinary excretion of the metabolite ranged from 2% to 13% of the inhaled dose. Hence there is more utility in measuring metabolites of budesonide for anti-doping screening. To clarify, this work demonstrates targeted LC–MS quantification for exposure/adherence assessment rather than metabolomics; under our review’s definition, a metabolomics investigation would additionally profile endogenous metabolite perturbations associated with the intervention (e.g., pathway-level changes) alongside the targeted measurements.

By uncovering drug-specific metabolic alterations, these approaches enhance anti-doping efforts and support the development of more personalized and time-resolved screening methods. This foundation sets the stage for exploring similar metabolomic strategies in detecting other classes of performance-enhancing drugs, such as anabolic steroids.

#### 2.2.2. Detection of Testosterone-Induced Metabolome Alterations

Testosterone and anabolic androgenic steroids (AASs) have been widely used in sports to enhance performance and muscle growth [67,68]. These substances are synthetic derivatives of testosterone, modified to enhance anabolic effects while minimizing androgenic properties [69]. Approximately 50% of administered testosterone undergoes conjugation via glucuronidation and sulfation [70], producing polar metabolites that facilitate urinary excretion [71]. These conjugated steroids serve as both direct biomarkers, such as testosterone glucuronide, and indirect biomarkers, such as altered ratios within the steroid profile, which are central to detecting exogenous testosterone administration [72]. In practice, urinary analyses rely on hydrolysis followed by GC-MS or LC-MS for long-term monitoring [73], whereas serum analyses increasingly measure intact conjugates directly by LC-MS/MS, providing complementary real-time metabolic insights [74].

Detection of testosterone doping is challenging due to its endogenous nature, relying on urinary steroid profile evaluation, carbon isotope ratio analysis and compound-specific 13C/12C analysis [69,75]. Athletes often use methods like “stacking” and “pyramiding” to maximize effects [68]. AAS abuse can lead to numerous side effects, including infertility, cardiovascular issues, liver damage, and psychological disturbances [68]. Anti-doping efforts have evolved to combat widespread use, as evidenced by state-sponsored doping programs in Germany and Russia [67].

To elucidate the efficacy of detecting metabolomic alterations in anti-doping, a review of selected cases on testosterone (and its derivatives) detection using metabolomic frameworks was conducted. These case studies highlight how metabolomic profiling, especially using advanced techniques such as non-targeted LC-MS workflows, enables the identification of specific metabolic changes associated with exogenous testosterone administration, rather than relying solely on standardized target analyte assays [76]. Such workflows, employing minimal preprocessing and normalization for urine specific gravity, can profile thousands of metabolic features, including both traditional and novel steroid conjugates post-administration [76]. Validation studies show the ability to discriminate between pre- and post-testosterone administration samples and to identify unknown urinary features correlated with testosterone exposure, offering sensitivity even amid high inter-individual variability [76]. The integration of these metabolomic markers offers promise for refining clinical and forensic biomarker panels and for extending retrospective detection windows for banned substances in doping control. Although these cases do not reflect the standard approach to testosterone abuse detection, they demonstrate how untargeted metabolomic analysis expands the detectable metabolic shifts, supports advanced data modeling, and contributes to the discovery of new biomarkers for anti-doping purposes.

Given the effects of androgenic steroids on metabolic pathways, a metabolomic approach to steroid abuse detection has a robust potential [77,78]. Studies have identified distinct metabolic profiles in high-power and high-endurance athletes, with changes in steroid biosynthesis, fatty acid metabolism, and oxidative stress markers [79]. Supraphysiologic testosterone administration profoundly alters steroid metabolism, generating specific metabolites detectable by MS. Approximately 50% of exogenous testosterone undergoes glucuronidation via UDP-glucuronosyltransferases (UGTs), yielding primary metabolites such as testosterone glucuronide (TG), androsterone glucuronide (AG), etiocholanolone glucuronide (EtioG), and dihydrotestosterone glucuronide (DHTG). These glucuronide conjugates are excreted primarily through MRP2 and MRP3 transporters in urine and bile [71,75]. These metabolites along with markers of anabolic pathway flux provide quantitative markers of exogenous testosterone use. Beyond steroid-specific measures, alterations in lipid, amino acid, and energy metabolism can further support integrated anti-doping strategies [77].

For example, a non-targeted LC-MS workflow profiled around 3000 urinary features (including glucurono- and sulfo-conjugated steroids), resolved pathways driving group separation despite high individual variability, and uncovered novel features linked to testosterone exposure [76]. Following a single transdermal testosterone dose, the method captured rises in testosterone and epitestosterone glucuronides with concomitant increases in glucocorticoid-pathway end-products and candidate markers (11-oxo-etiocholanolone G, 11-oxo-androsterone G, and 11β-hydroxy-androsterone/etiocholanolone G or their 6β-OH forms), and it revealed additional unidentified glucuronidated features as evidence of androgenic steroid administration [76]. Moreover, ultra-high pressure liquid chromatography coupled with mass spectrometry has been used to quantify urinary steroids and investigate steroid metabolism after testosterone administration [30]. Combining carbon isotope ratios with urinary concentrations of testosterone metabolites has shown promise in extending detection windows for steroid hormone administrations [80]. Additionally, a single-run UHPLC-MS/MS method has been developed to simultaneously quantify endogenous steroids and their phase II metabolites in serum, offering potential advantages for detecting testosterone abuse in females and individuals with UGT2B17 enzyme deletion [81]. These advanced techniques aim to improve the sensitivity and specificity of steroid abuse detection in sports.

Recent studies have utilized high-resolution mass spectrometry coupled with liquid chromatography to identify novel biomarkers in urine and serum samples [31,81,82]. This method has successfully revealed potential markers, such as 1-cyclopentenoylglycine, which is linked to the metabolism of testosterone cypionate [31]. Researchers have also explored the impact of genetic polymorphisms on steroid metabolism, highlighting the importance of addressing inter-individual variability [82]. In addition, the application of steroidomics in serum samples has shown promise for longitudinal monitoring of steroid hormones [81]. Furthermore, the transfer of non-targeted metabolomics findings to targeted biomarker strategies has been demonstrated, allowing for more widespread implementation in routine doping control [83]. These findings highlight the method’s adaptability and promise for long-term monitoring and individualized testing strategies, reinforcing its potential as a cornerstone of future anti-doping protocols.

## 3. AI/ML in Anti-Doping

The integration of AI and ML into anti-doping strategies marks a transformative phase in the detection and deterrence of performance-enhancing drug (PED) use in sports. Traditional anti-doping methodologies have relied on routine biological and analytical testing [84], which, although essential, are often reactive and limited in sensitivity, especially when facing increasingly sophisticated doping methods [16]. AI and ML offer a paradigm shift—introducing predictive, data-driven approaches that enhance the efficiency and accuracy of anti-doping efforts. AI techniques effectively process vast amounts of biological and behavioral data, identifying patterns and anomalies associated with doping, while reducing false positives and false negatives [16]. The technology also allows for real-time analysis, adaptation to emerging doping strategies, and integration with other innovative tools such as blockchain, big data analytics, and advanced imaging techniques, thereby improving the reliability and efficiency of doping tests [16]. These innovations not only make doping detection more cost-effective and non-invasive but also strengthen the integrity of sports competitions by providing more precise and timely results, ultimately contributing to fairer play and athlete health protection [16]. Through the development of predictive models, the integration of multi-omics data, and the application of AI in specific case studies, sports science is leveraging computational power to outpace doping innovations and better safeguard the integrity of competitive athletics.

### 3.1. Predictive Models

Predictive modeling is one of the most prominent applications of AI and ML in anti-doping. Both supervised and unsupervised learning techniques are being increasingly employed to identify athletes at risk of doping, optimize resource allocation, and detect physiological anomalies in longitudinal data [1].

#### 3.1.1. Supervised Learning

Supervised learning methods use labeled data—such as confirmed doping cases and corresponding biomarker profiles—to train predictive models capable of identifying suspicious patterns. Algorithms like XGBoost (Extreme Gradient Boosting) and Multi-Layer Perceptrons (MLPs) have demonstrated strong performance in such tasks [85].

XGBoost, a decision-tree-based ensemble algorithm, is particularly adept at handling structured data and complex, nonlinear interactions among variables [86]. It excels in classifying athletes based on features extracted from biological parameters such as hemoglobin levels, reticulocyte counts, and other biomarkers included in the ABP [13]. XGBoost demonstrated superior classification performance, achieving an accuracy of 92%, which exceeds that of Support Vector Classification (71%) and Random Forest (85%) [13]. The state-of-the-art (SOTA) model was employed as a baseline for performance comparison. In terms of sensitivity, XGBoost outperformed the SOTA model with a rate of 68%; however, it exhibited a 3% lower specificity relative to the SOTA benchmark [13]. The model’s capability to rank feature importance also aids anti-doping experts in understanding which variables are most predictive of suspicious activity.

Multi-Layer Perceptrons (MLP), on the other hand, are a class of feedforward artificial neural networks that can model highly complex relationships in data [85,87]. MLPs are well-suited for high-dimensional input spaces, such as those generated by longitudinal athlete monitoring systems. In a study conducted by Ryoo et al., MLP demonstrated strong utility within an ensemble framework for identifying potential doping among elite female weightlifters [10]. Optimized via grid search and validated using five-fold cross-validation, the MLP effectively captured complex, nonlinear patterns in high-dimensional performance and demographic data [10]. Its integration with XGBoost contributed to the ensemble’s overall predictive accuracy of 87.5% and an AUC-ROC of 0.783 [10]. Although the MLP’s individual feature contributions were not explicitly analyzed, its inclusion enhanced model robustness, highlighting the value of neural network architectures in supporting data-driven, proactive anti-doping strategies [10]. Given enough labeled data, MLPs can learn complex patterns that may indicate subtle deviations in physiological markers consistent with doping. However, one limitation of supervised models is their dependence on labeled data, which is often scarce in the anti-doping context due to underreporting and the clandestine nature of doping behavior. In contrast, unsupervised learning methods do not rely on labeled data, making them ideal for exploratory tasks and anomaly detection [1]. Techniques such as clustering (e.g., k-means, DBSCAN) and dimensionality reduction (e.g., PCA, t-SNE) help identify unusual patterns or outliers in athlete data without prior knowledge of what constitutes a doping event [1]. These methods can automatically flag suspicious ABP profiles by comparing an individual athlete’s trajectory against normative population baselines, facilitating a proactive approach to investigations [1].

#### 3.1.2. Unsupervised Learning

Having detailed supervised approaches, we next consider unsupervised methods that do not rely on labeled data. These techniques are particularly valuable for exploratory analysis, anomaly detection, and pattern recognition where definitive examples of doping are unavailable [1]. Unsupervised algorithms, such as clustering methods (e.g., k-means, DBSCAN) and dimensionality reduction techniques (e.g., PCA, t-SNE), are used to uncover hidden structures in the data [88]. For instance, these methods can identify outliers in an athlete’s ABP profile by comparing their data trajectory to normative population baselines. This enables flagging of atypical patterns without predefined categories of “doped” vs. “clean,” making it a proactive tool in long-term surveillance.

One of the most impactful advancements in this area is the development of AI-enhanced ABP models, particularly for the detection of EPO. In EPO detection, AI integration allows for better discrimination between endogenous fluctuations and pharmacological intervention [16]. EPO’s short half-life and the presence of biosimilar agents have made it notoriously difficult to detect [89]. AI models trained on proteomic and hematologic profiles can learn nuanced differences in molecular markers or response curves associated with synthetic EPO administration. Additionally, predictive modeling can incorporate genetic and transcriptomic data, identifying athletes with an unusual ability to generate endogenous EPO—thus refining the specificity of detection. A recent article showcased the development of a ML-based model capable of accurately distinguishing between endogenous and synthetic EPO in blood samples [16]. Unlike conventional methods that require large sample volumes and time-intensive analyses, this AI model demonstrated high performance using smaller blood volumes [16]. Trained on datasets comprising both natural and synthetic EPO, the model achieved enhanced accuracy in classifying previously unseen samples [16]. Notably, the AI model outperformed traditional techniques in sensitivity and specificity, successfully detecting synthetic EPO even in cases where standard assays failed [16]. These findings highlight the potential of AI to significantly enhance EPO detection, offering a more efficient and precise tool for anti-doping applications, and supporting the broader integration of AI technologies in the fight against blood doping.

The implementation of such predictive models significantly enhances the efficiency of testing programs. By narrowing down the pool of athletes for in-depth testing, these models enable anti-doping agencies to allocate their limited resources more effectively, increasing the probability of catching violators while minimizing unnecessary testing of clean athletes. As such, AI and ML stand as indispensable tools in the modernization of anti-doping operations.

### 3.2. Multi-Omics Integration

The future of anti-doping lies not only in predictive algorithms but also in the holistic integration of multi-omics data. Multi-omics refers to the simultaneous analysis of data from various biological layers, such as genomics, transcriptomics, proteomics, metabolomics [90], and more, alongside biometric and behavioral information. This integrative approach acknowledges the complexity of biological systems and seeks to build a more complete picture of an athlete’s physiological state. Omics enable precise and reproducible profiling of genes, transcripts, proteins, and metabolites. Genomics and transcriptomics have been explored for gene doping detection, while proteomics has shown promise in identifying hormone doping [27].

Among the most promising applications is the fusion of metabolomic, biometric, and behavioral data using AI. Metabolomics has emerged as a promising tool in anti-doping efforts, enabling the identification of metabolic perturbations in athletes that may indicate the use of prohibited substances [27]. By serving as a high-throughput screening approach, metabolomics can highlight individuals exhibiting atypical metabolic profiles, potentially resulting from doping practices [27]. Within this field, steroidomics—a specialized branch focusing on the comprehensive analysis of steroidal metabolites—has already demonstrated utility in characterizing steroidal signatures and detecting anabolic androgenic steroid (AAS) misuse [27]. Steroidomics employs a semi-targeted strategy to identify direct biomarkers indicative of AAS administration, offering enhanced sensitivity and specificity compared to conventional methods [27]. When integrated with biometric data such as sleep patterns, heart rate variability, or training loads—often collected through wearable devices—ML models can identify physiological discrepancies that may signal illicit drug use [91,92].

Behavioral data, including travel schedules, competition frequency, and even psychological metrics such as mood or perceived exertion, can further refine risk profiles. Athletes often alter their routines in anticipation of doping or testing windows, and such behavioral shifts can be captured and analyzed using advanced AI algorithms.

A recent study introduced SACNN, a self-attention-based convolutional neural network that detects doping-related fraud, such as sample swapping, by analyzing spatio-temporal behavioral patterns in athletes’ longitudinal profiles [93]. Enhanced with adversarial training, SACNN outperforms existing models and reduces reliance on costly DNA testing, offering an effective and scalable tool for anti-doping screening [93]. Integrating multi-omics and behavioral data transforms the ABP from a relatively narrow hematological surveillance tool into a comprehensive digital twin of the athlete, capable of flagging even subtle, indirect signs of doping.

Ultimately, the promise of multi-omics integration lies in its potential to detect doping indirectly, not by identifying the substance itself, but by modeling the body’s response to it. This shift from forensic toxicology to systems biology represents a foundational transformation in anti-doping research.

### 3.3. Case Studies

To demonstrate the potential of AI and machine learning in detecting doping practices, we present select case studies from the literature that highlight their practical applications and growing effectiveness. It is worth noting that these cases present experimental reports, not current standard methods in doping control.

Several recent case studies highlight the practical utility and evolving performance of AI-based anti-doping tools. One such example involves the use of ensemble models in elite female weightlifters [10]. The study demonstrated that AI-powered ensemble models, particularly the combination of XGBoost and MLP, were effective in predicting doping suspicion among elite female weightlifters based on ABP data and performance metrics [10]. The optimal ensemble model achieved an accuracy of 87.5%, an AUC-ROC of 0.783, and an F1 score of 0.645 on the test dataset. It notably identified approximately 66.7% of sanctioned athletes from the 2008 Olympics, 56.3% from 2012, and 25% from 2016, while also maintaining a reasonable rate of correctly classifying non-doped athletes [10]. The ensemble learning approach, which integrated multiple machine learning algorithms—including random forests, gradient boosting machines, support vector machines, and neural networks—yielded a classification accuracy of 53.8% when broadly applied across all models [10]. Although modest, this performance reflects the inherent challenges of detecting doping in the absence of definitive ground truth and highlights the potential of AI-based analysis of ABP data to enhance proactive anti-doping efforts in elite sports.

A randomized controlled trial (RCT) was conducted on recreational athletes residing in Australia (16 women and 33 men) and China (12 women and 12 men) who were randomly assigned to receive either recombinant human erythropoietin (rhEPO) or placebo over a 25-day period [94]. This study investigated the reliability of five indirect hematologic markers—hematocrit (Hct), reticulocyte hematocrit (RetHct), percentage of macrocytes (%Macro), serum erythropoietin (EPO), and soluble transferrin receptor (sTfr)—as indicators of rhEPO use in athletes [94]. Participants were randomly assigned to receive either rhEPO or placebo over 25 days, with blood profiles monitored during and up to four weeks post-administration [94]. The results demonstrated consistent and predictable changes in these markers across both populations, confirming their utility in detecting current or recent rhEPO use regardless of ethnicity [94]. These findings support the development of an indirect blood test that can effectively identify and deter rhEPO abuse in athletes.

Another study aimed to develop a robust indirect method for detecting blood doping with rhEPO by leveraging statistical analysis and ML algorithms [95]. Traditional laboratory-based tests for rhEPO detection are often expensive, time-consuming, and logistically challenging; hence, the study sought to enhance detection capabilities by analyzing hematological parameters through AI-driven approaches [95]. By integrating advanced computational techniques, the researchers aimed to improve the accuracy, sensitivity, and overall reliability of doping detection, thereby supporting sports authorities in maintaining fair competition—particularly in contexts where direct testing is not feasible [95]. The findings demonstrate that machine learning models, particularly the XGBoost algorithm, are effective in identifying rhEPO-induced blood doping using hematological markers [95]. The XGBoost model achieved an accuracy of 92%, a sensitivity of 68%, and an F1-score of 0.95, outperforming conventional methods and other ML models such as Support Vector Classifier (SVC) and Random Forest (RF) [95]. These results underscore the utility of ensemble and boosting techniques in indirect doping detection.

These case studies demonstrate the real-world applicability and evolving accuracy of AI systems in anti-doping. They also highlight the importance of transparency, model interpretability, and ethical considerations. Given the potentially career-altering consequences of false positives, it is essential that these AI models are used to support, rather than replace, human judgment. Explainable AI (XAI) tools are thus being developed in parallel to provide rationales for algorithmic decisions, ensuring that athletes and regulatory bodies alike can understand and trust the outputs.

In a nutshell, the application of AI and ML in anti-doping has already begun to revolutionize how cheating in sport is detected and deterred. Through predictive modeling, multi-omics integration, and real-world case studies, these technologies are enhancing the precision, proactivity, and personalization of anti-doping strategies. While challenges remain in terms of data availability, ethical use, and algorithmic transparency, the trajectory is clear: AI will play a central role in preserving the fairness and integrity of sport in the 21st century.

## 4. WADA Technical Framework and Its Impact on Metabolomics and Machine Learning Approaches

The integration of metabolomics and ML into anti-doping research and practice is necessarily guided by the WADA technical documents, particularly those regulating the EAAS module of the ABP. These documents establish stringent requirements for analytical performance, biomarker selection, and statistical evaluation, which in turn shape how emerging metabolomics- and ML-based strategies are developed and validated.

WADA’s Technical Document for EAAS (TD2021EAAS) specifies the markers of the urinary steroid profile (e.g., testosterone, epitestosterone, androsterone, etiocholanolone, 5α- and 5β-androstanediol), defines minimum analytical requirements, and sets ratio-based thresholds such as the T/E ratio of 4:1 as an initial decision limit [54]. This framework requires that any metabolomics-derived biomarkers be interpreted in the context of validated steroid conjugates and their ratios. Untargeted metabolomics approaches therefore must demonstrate precision, reproducibility, and longitudinal interpretability that meet WADA’s laboratory accreditation standards [96].

Similarly, the ABP operating guidelines mandate the use of adaptive Bayesian models to detect individual deviations from expected steroid profiles [17]. This has direct implications for ML-based approaches, as algorithms must integrate within-subject variability, population priors, and statistical thresholds defined by WADA. For example, while untargeted LC/HRMS and GC/HRMS workflows can discover novel metabolites, these candidates must ultimately align with WADA’s criteria for sensitivity, specificity, and interpretability to be admissible in doping adjudication [20,57].

In practice, this alignment means that metabolomics and ML-driven anti-doping strategies are not purely exploratory but rather anchored to the EAAS framework and broader WADA technical documents. Biomarker discovery (e.g., novel steroid conjugates or metabolic perturbations) and statistical thresholds are always interpreted against WADA’s validated analytical targets, confirmatory decision limits, and IRMS confirmation protocols [42,97]. As such, WADA’s technical documents act as both a constraint—ensuring harmonization and legal defensibility—and an enabler, providing a regulatory scaffold for translating innovations in metabolomics and ML into routine anti-doping practice.

## 5. Challenges and Future Directions

It is worth mentioning that, while advanced ML-integrated/metabolomics strategies for anti-doping are highlighted in this work, these approaches remain largely in the research and validation stages; routine anti-doping practice in accredited laboratories continues to rely on established targeted analytical methods [98].

### 5.1. Metabolomics Limitations

Metabolomics face significant challenges in standardization and data processing. Biological variability, analytical inconsistencies, and lack of unified protocols hinder data comparability across studies and laboratories [99]. Researchers have identified pre-analytical, intra-analytical, and post-analytical sources of variation, emphasizing the need for standardized methods and quality control procedures [99,100]. Furthermore, biological variation in human samples can be substantial, with median coefficients of variation of 35% for cerebrospinal fluid and 46% for plasma [101]. Factors like circadian rhythms can greatly influence metabolite levels, which requires well-thought experimental designs to account for their effects [102].

To address these issues, method validation strategies are crucial in non-targeted metabolomics, including careful sample selection, pre-treatment, and data analysis [103]. MS-based techniques have become preferred methods for metabolome characterization, but progress is hindered by a lack of standardized procedures and difficulties in metabolite identification [100]. Improving sample collection, preparation, and mass spectrometry analysis can enhance metabolite coverage and identification [100]. The Metabolomics Society has initiated efforts to define standardized reporting structures for metabolomic studies, addressing aspects such as study design, chemical analysis, and data processing [104]. As the field generates increasing amounts of data, developing suitable analysis methods and validation procedures becomes crucial [105].

There are limitations to metabolomics that are specific to anti-doping. These include challenges in data processing, metabolite identification, and distinguishing doping-related changes from natural variations in metabolism [106]. Moreover, the complexity of biological systems and the vast number of metabolites present additional obstacles [107]. Despite these constraints, ongoing research aims to refine metabolomics techniques for anti-doping applications. Combining high-resolution MS with chemometric tools has shown potential for differentiating changes due to athletic activities from those caused by prohibited substances [108]. As the field evolves, metabolomics may become a valuable addition to the anti-doping toolbox. Addressing the aforementioned limitations through unified protocols and standardization efforts is essential for advancing metabolomics research and enabling accurate and robust use for anti-doping purposes.

Additionally, Metabolomics data are inherently influenced by several confounding factors, including circadian rhythms, dietary intake, inter- and intra-individual biological variation, and technical artifacts introduced during sample collection, preparation, or analysis [109,110]. These sources of variability can obscure true drug-related metabolic signatures and reduce the reliability of biomarker discovery. ML techniques offer powerful strategies to address these challenges: unsupervised approaches such as anomaly detection can identify outliers or unexpected shifts in the data, while statistical and ML-based batch correction and normalization methods [111,112] can mitigate technical variability across analytical runs. Furthermore, ML algorithms can incorporate covariates (e.g., time of day, dietary records) into predictive models, allowing for partial disentanglement of biological confounders from drug-induced changes [113]. By systematically detecting, flagging, and correcting for such sources of variation, ML can enhance both the robustness and translational validity of metabolomics-based anti-doping applications.

Finally, although promising, large-scale metabolomics in anti-doping is operationally demanding beyond instrument selection. Pre-analytical control (standardized sampling timepoints, collection materials, nutrition/training status, illness/altitude/supplement use, and strict chain-of-custody) is critical to minimize biological and logistical variance. Post-analytical rigor is likewise required: matrix-appropriate stabilization and storage (temperature, minimized freeze–thaw), randomized batch design, system-suitability checks, pooled QCs and blanks, continuous signal-drift monitoring and correction, and stringent feature curation/annotation. These steps materially affect data quality, comparability, and interpretability. In addition, lab-to-lab harmonization and transparent data governance are essential for legal defensibility. Finally, ML models are susceptible to leakage and distribution shift; robust pipelines should use nested cross-validation, calibration, external/multicenter validation, and pre-registration to ensure generalizability and reduce false-positive risk [17,114,115,116].

### 5.2. AI/ML Barriers

Artificial intelligence and machine learning offer promising tools for anti-doping efforts but face several limitations. On the technical side, data-related challenges persist, including small and imbalanced datasets, algorithmic bias, and inappropriate evaluation metrics [117]. Broader computational issues, such as combinatorial complexity and limitations in inference, also constrain AI/ML capabilities in real-world doping detection [118]. Despite these barriers, AI holds the potential to enhance the speed and efficiency of anti-doping checks [119]. However, progress is impeded by the limited availability of large-scale, longitudinal performance and biomarker datasets.

Beyond data and technical challenges, implementation of AI in anti-doping raises a distinct set of ethical questions. What rights do athletes have regarding how their biometric and performance data are collected, stored, and used by AI systems? Can AI-based profiling lead to unfair suspicion or targeting of certain individuals or demographics? What safeguards exist to ensure that AI does not reinforce existing racial, gender, or socioeconomic biases in sports governance? If an AI model wrongly flags an athlete, who is held accountable—the developer, the data provider, or the governing body?

These concern not only the moral standing of current anti-doping systems but also the broader social implications of AI-based surveillance and profiling [120]. AI technologies offer promising solutions for removing accessibility barriers for people with disabilities, yet ethical concerns such as bias, privacy, and social acceptability must be addressed [121]. In sports, AI applications raise ethical issues related to fairness, transparency, privacy, and accountability [122]. While AI has the potential to enhance accessibility for individuals with disabilities, algorithmic biases may perpetuate discrimination against marginalized groups [123]. These studies emphasize the need for careful consideration of ethical implications when implementing AI in sports and accessibility contexts, and sequentially, the importance of developing tailored ethical frameworks and global standards for AI regulation.

As a potential solution, development of open-access AI tools is crucial for global anti-doping agencies in combating doping in sports. Choudhary and Mehta highlight AI’s potential to enhance the efficiency of doping tests, making them faster and more cost-effective [119]. Their subsequent work discusses AI’s role in identifying drug trafficking on the dark web, which poses a significant threat to young athletes [124]. Kelly et al. emphasize the need for advanced machine learning techniques to identify athletes at high risk of doping, suggesting that these methods can optimize resource allocation for anti-doping organizations [125]. Holz and Robertson provide historical context, noting the establishment of the WADA and the importance of intelligence gathering in anti-doping efforts, particularly through collaboration with law enforcement [126]. To maximize the potential of AI in anti-doping efforts, it is crucial to address each of the previously mentioned limitations and develop more accessible and enriched data systems [10].

### 5.3. Emerging Opportunities

Despite limitations and challenges, the utility of metabolomics and AI for doping detection remains substantial with many potential future applications. For example, sport-specific AI models for anti-doping present promising findings. Recent research highlights the application of artificial intelligence in anti-doping efforts within weightlifting. Ryoo et al. introduced an AI-driven APP to identify doping suspicions among elite female weightlifters, achieving a 53.8% prediction rate for sanctioned athletes [10]. Novatchkov and Baca demonstrated the potential of AI techniques in evaluating exercise performance, using AI-based pattern recognition for optimizing training and preventing doping [127]. It should be noted that current studies often rely on small sample sizes, underscoring the need for multicenter and longitudinal datasets to ensure translational robustness of metabolomics-based anti-doping approaches.

Although MS-based metabolomics are primarily laboratory-based, experimental advances in portable MS now enable rapid, preliminary anti-doping screening in the field. Hendricks et al. describe a backpack-mounted miniature mass spectrometer capable of detecting chemical warfare agents, illicit drugs, and explosives at nanogram levels, demonstrating its potential for in situ analysis [128]. Wells et al. suggested a mobile laboratory mass spectrometer that integrates various ionization techniques, enabling rapid detection of compounds relevant to quality control and forensic applications [129]. Additionally, Heaney et al. showcase a compact mass spectrometer for real-time monitoring of exhaled volatiles, indicating the feasibility of portable systems for breath analysis in detecting doping substances [130]. While miniaturized MS is advancing and despite its promising potential, MS-based metabolomics remains a laboratory workflow: athlete samples are analyzed in WADA-accredited labs using validated targeted methods and IRMS, whereas portable MS is limited to research or narrow field screening (e.g., seized materials or pre-analytical triage) and currently lacks the validation, chain-of-custody, and confirmatory capability required for real-time, in-competition testing.

**Figure 1:**
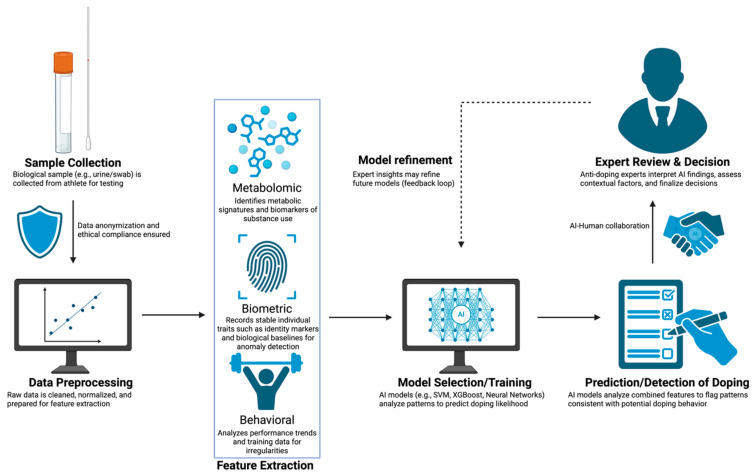
Integrated Workflow of Metabolomics and AI in Modern Anti-Doping Strategies.

In summary, integrating advanced metabolomics and AI methods into anti-doping efforts, particularly through sport-specific AI models and portable MS technologies, offers promising capabilities to revolutionize doping detection, paving the way for enhanced accuracy, real-time monitoring, and proactive interventions in competitive sports. Figure 1 provides a conceptual overview of the integrated workflow combining advanced metabolomics, biometrics, and AI/ML for modern anti-doping detection and decision-making.

## 6. Conclusions

The merging of metabolomics and ML marks a transformative turning point in anti-doping science. By advancing past traditional compound-centric detection, metabolomics provides a comprehensive, systems-level view of biochemical modifications linked to doping, capturing both direct and indirect biomarkers. The integration of AI and ML enables the interpretation of complex, high-dimensional datasets, greatly improving the accuracy and efficiency of doping detection protocols. Real-world case studies exhibit the potential of advanced computational tools to identify subtle metabolic alterations, allowing for proactive, personalized athlete monitoring. However, the impact of these approaches relies on standardized protocols, available and diverse datasets, and rigorous validation of analytical and computational methods. To take full advantage of metabolomics and AI in anti-doping, ongoing collaboration among analytical chemists, clinicians, data scientists, and regulatory authorities is crucial. Such multidisciplinary efforts will be essential to generate robust, ethical, and universally applicable anti-doping measures, ultimately safeguarding both sporting integrity and athlete health.

## Figures and Tables

**Table 1:** Comparative Overview of Analytical Platforms for Doping Material Detection.

Analytical Platform	Key Features	Advantages	Limitations	Targeted/Untargeted	References
LC-MS/MS (Liquid Chromatography–Tandem Mass Spectrometry)	Most frequently used in doping screening	- High sensitivity, precision, and reproducibility - Effective for quantifying low-abundance metabolites - Discriminates structurally similar metabolites (improved specificity)	Context-dependent sample preparation *: for many matrices/targets, simple dilute-and-shoot or protein precipitation is sufficient; escalation to SPE/LLE or additional cleanup is used for complex matrices, isobaric interferences, or very low-abundance analytes	Primarily targeted (with semi-untargeted applications)	[26,40]
NMR Spectroscopy (Nuclear Magnetic Resonance)	Non-destructive, highly reproducible; excellent for structural elucidation	- Quantitative, non-destructive, highly reproducible - Excellent for structural elucidation - Suitable for detecting broad metabolic shifts	- Lower sensitivity than MS platforms - Less effective for low-abundance metabolites	Primarily untargeted	[48,49]
High-Resolution MS Techniques (e.g., Orbitrap, FT-ICR/MS, QTOF-MS)	Used in untargeted metabolomics and biomarker discovery	- High accuracy and resolution - Enables detection of novel or unknown metabolites - Differentiates physiological vs. doping-induced changes	- Higher cost - Complex data analysis	Both targeted and untargeted	[7,50,51,52,53]
GC-MS/MS	Gas chromatography with triple-quadrupole MS/MS; often preceded by enzymatic hydrolysis, LLE/SPE, and derivatization (e.g., TMS)	High selectivity/sensitivity for volatile/derivatized analytes; extensive method maturity in WADA labs; aligns with MRPL guidance for many classes	Requires derivatization for many steroids; less suitable for very polar/thermolabile metabolites; matrix-dependent prep	Targeted (primary); limited untargeted	[26,45]
GC-C-IRMS (Isotope-Ratio MS)	GC separation coupled to on-line combustion and IRMS for δ^13^C of target steroids vs. endogenous references	Distinguishes synthetic from endogenous steroid origin; mandated confirmatory tool after suspicious steroid profiles; strong legal defensibility	Specialized instrumentation; higher sample/analysis time per target; requires sufficient analyte abundance and clean chromatographic resolution	Targeted confirmatory	[42,43,44]

* LC-MS/MS preparation varies by matrix/target. Many methods use minimal prep (e.g., dilution or protein precipitation). More elaborate workflows (e.g., SPE/LLE, additional cleanup) are reserved for complex matrices, isobaric interferences, or ultra-trace quantitation.

## Data Availability

No new data were created or analyzed in this study.
